# “Envbiotics” -- a novel framework for microbiota-targeted therapeutic strategies in type 2 diabetes mellitus

**DOI:** 10.3389/fendo.2026.1878037

**Published:** 2026-07-08

**Authors:** Wenxiao Wang, Long Xu, Ping Leng, Man Jiang, Xiaomin Xing, Mingchen Cao, Fanbo Jing

**Affiliations:** The Affiliated Hospital of Qingdao University, Qingdao, China

**Keywords:** envbiotics, gut microecosystem, host-microbiota interaction, insulin resistance, type 2 diabetes mellitus

## Abstract

Imbalance of the gut microbiota is an important trigger for insulin resistance in type 2 diabetes mellitus(T2DM). Microbiota-targeted therapies have gradually become an emerging research direction for treating T2DM. However, exogenous strain interventions (supplementing probiotics and fecal microbiota transplantation), endogenous optimization of the gut microbiota (supplementing prebiotics, synbiotics, and postbiotics), and phage therapy focus on “directly introducing microorganisms,” “feeding microorganisms,” or “directly utilizing microbial components.” These approaches cannot cover active substances that target the host as the core and regulate the intestinal microenvironment in a non-nutritional manner, presenting conceptual limitations. In this context, this paper proposes the concept of “Envbiotics, ” defined as substances that target the host as the core, optimize the intestinal microenvironment through their own or host metabolites, and directly or indirectly regulate the structure and function of the microbiota in a non-nutritional manner, thereby improving host metabolism and health. Typical evidence, including berberine, urolithin A, plant exosomes, and special targeted delivery technologies, is used to elucidate its mechanism of action. Envbiotics not only fill the gaps in the existing classification system but also provide novel insights for the development of new drugs targeting microbial intervention in T2DM.

The gut microbiota plays an important role in maintaining metabolic homeostasis in the body. In patients with type 2 diabetes mellitus(T2DM), the overall level of butyrate-producing Firmicutes bacteria is generally reduced, which can affect intestinal barrier function and systemic inflammatory responses, ultimately exacerbating insulin resistance. Microbiota-targeted therapies have gradually become an emerging research direction for treatingT2DM(see **Figure 1**), such as probiotic supplementation and Fecal Microbiota Transplantation (FMT). However, exogenous strain interventions have limitations, including low glucose disposal response rates and significant individual differences (1). Therefore, targeting the optimization of the structure and functional activity of the endogenous microbiota may hold greater application prospects, such as supplementing with prebiotics, synbiotics, and postbiotics. Prebiotics are substrates selectively utilized by host microorganisms, which must have a clear and single chemical structure (2). Synbiotics are mixtures containing live microorganisms and substrates selectively utilized by host microorganisms (3). Postbiotics are non-viable bioactive substances obtained from probiotics and their metabolites after specific processing (4). All three are intestinal microecological regulators beneficial to host health (see **Table 1**). Additionally, phage therapy achieves precise elimination by specifically lysing target bacteria while minimizing damage to the gut microbiota, offering a highly promising alternative for the precise treatment of diabetic foot ulcers and diabetic periodontitis (5). However, clinical translation still faces challenges such as insufficient evidence, incomplete regulation, and unstable transportation.

**Figure 1:**
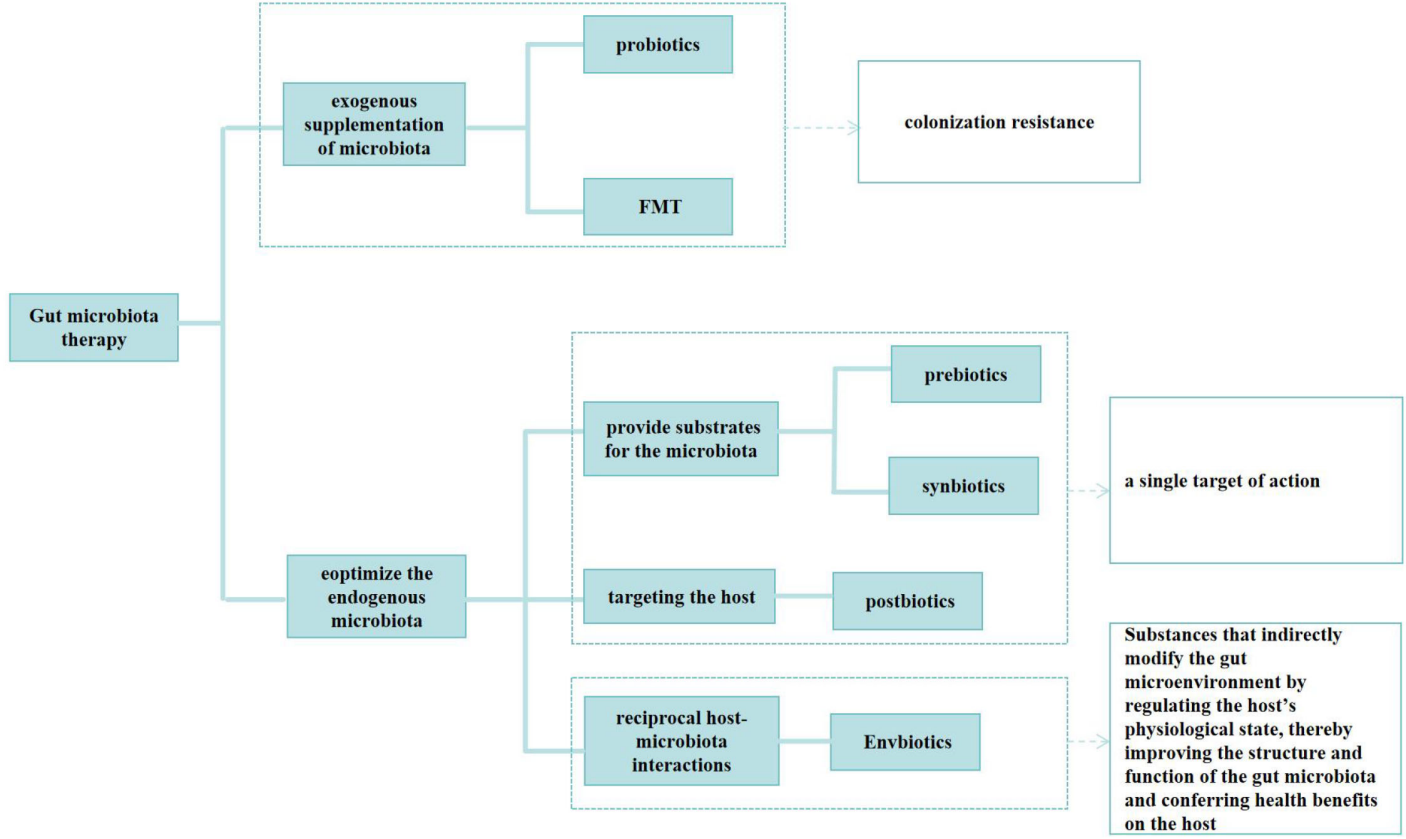
Classification of gut microbiota management strategies.

**Table 1:** Comparison of microbiota-targeted intervention method.

Item	Probiotics	Prebiotics	Synbiotics	Postbiotics	Envbiotics	FMT	Phage therapy
Definition	Live microorganisms that, when administered in adequate amounts, confer a health benefit on the host	a substrate that is selectively utilized by host microorganisms conferring a health benefit	A mixture comprising live microorganisms and substrate(s) selectively utilized by host microorganisms that confers a health benefit on the host.	A postbiotic is a preparation of inanimate microorganisms and/or their components that confers a health benefit on the host.	With the host as the core target, optimize the gut microbiome through its own or host metabolites, directly or indirectly regulate the flora structure and function in a non-nutritional manner, thereby improving host metabolism and health.	Transplant the functional fecal microbiota from healthy individuals into the patient’s intestine to restore gut microbiota homeostasis, thereby achieving the goal of treating diseases.	Achieve precise elimination by specifically lysing target bacteria.
Substance Property	Live microbial preparation	Cannot be absorbed or utilized by the human body, but can be selectively utilized by beneficial bacteria, and has a clear, single chemical structure.	Live bacteria and substrate compound preparation	Inactivated bacteria, metabolites, cell wall components, etc.	Active substances that can act on host tissues and regulate the physicochemical conditions of the intestinal environment.	Complete fecal mixed microbiota	Specific lytic bacteriophage virus
Mode of Action	Supplement probiotics	Provide nutrients for probiotics	Supplement probiotics while providing substrates for probiotics to promote colonization.	Regulating host immunity, intestinal barrier, etc.	Regulating the host’s physiological state to modulate the intestinal microenvironment	Reconstructing the composition of the recipient’s gut microbiota	Targeting specific bacteria - Adsorption - Injection - Replication - Lysis
Regulation method	Regulation of microbial community structure	Microbiota nutrient supply regulation	Microbial community structure + nutritional composite regulation	Host immunity regulation	reciprocal host-microbiota interactions	Reconstruction of the overall microbial community	Single pathogenic bacteria targeted elimination
Typical representative	Bifidobacterium	Inulin	Bifidobacterium oligosaccharides	Short chain fatty acids	mulberry leaf water extract	Fecal microbiota freeze-dried capsules (6)	TP-102 (7)

The above concepts focus on “directly introducing microorganisms,” “feeding microorganisms,” or “directly utilizing microbial components.” However, many natural active compounds do not regulate the microbiota through direct supplementation or nutritional supply. Instead, they target the host as the core, optimizing the intestinal microenvironment through their own or host-derived metabolites, directly or indirectly regulating the structure and function of the microbiota in a non-nutritional manner, thereby improving host metabolism and health. Based on this, our team first proposed the concept of “Envbiotics” (8) to supplement the shortcomings of existing concepts. Envbiotics provide a new strategy for microbial-targeted intervention in T2DM. Envbiotics should have the following characteristics: (1) indirectly altering the intestinal microenvironment by regulating the host’s physiological state, thereby improving the structure and function of the gut microbiota; (2) confer health benefits on the host; (3) with or without the role of nutritional supply to gut microorganisms; (4) exerting the above effects after being metabolized by the host’s gut microbiota, or it can act directly.Preparations that serve only as nutritional substrates for gut microorganisms, live microbial preparations, or non-living microbial preparations do not fall within the scope of Envbiotics.

Many bioactive natural compounds require metabolic transformation by various enzymes (such as β-glucosidase, nitroreductase, 7α-hydroxylase, etc.) produced by the gut microbiota. However, these enzymes are not exclusively produced by beneficial bacteria, so such compounds cannot be classified under traditional microecological modulators. Taking berberine as an example, the opportunistic pathogen *Escherichia coli* can convert berberine into oxyberberine, while another opportunistic pathogen *Enterococcus faecium* promotes berberine transformation by participating in nitroreduction reactions. After metabolism by non-probiotic bacteria, the hypoglycemic and anti-inflammatory activities of berberine are enhanced (9). Non-universal metabolites produced through non-probiotic metabolic pathways from specific substrates (certain natural drug components), i.e., differentiated metabolites generated in specific environments, also seem unsuitable for inclusion in the category of traditional microecological modulators. For instance, urolithin A relies on the metabolism of the specific substrate ellagic acid by specific gut microbiota to be produced. However, the urolithin components generated through metabolism are no longer metabolized by the gut microbiota but directly act on the host’s immune system and regulate blood glucose levels. Direct supplementation of urolithin A can also achieve the effects of modulating the gut microbiota and blood glucose levels (10). Although there is no direct data on the excretion pathway of urolithin A, based on its chemical properties and the metabolic patterns of similar metabolites, it is speculated that urolithin A is absorbed by intestinal epithelial cells, enters the systemic circulation to exert its effects, and is no longer processed through intestinal pathways, ultimately being excreted mainly via the kidneys in urine.

Some natural active compounds can be directly utilized by the gut microbiota and also act on the host to improve metabolism, exerting a dual effect. Therefore, they cannot be classified within the traditional category of microecological regulators. For example, mulberry leaf water extract (MLWE) can regulate the expression of pro-inflammatory factors through the adenosine 5’-monophosphate-activated protein kinase (AMPK) pathway, alleviate insulin resistance in T2DM, and simultaneously increase the abundance of beneficial bacteria while reducing the abundance of harmful bacteria (11).

In addition, certain special formulations can alter the absorption characteristics and bioavailability of the original active ingredients, enabling them to act through host cell-targeted uptake rather than microbial nutrient utilization pathways. For example, exosome-like vesicles (ELVs) are biological macromolecular complexes rich in DNA, RNA, and proteins, primarily taken up and utilized by host intestinal immune cells, and cannot be directly used as nutritional substrates by gut microorganisms; therefore, they do not belong to traditional gut microbiome modulators. For example, tangerine-peel-derived ELNs (TNVs) significantly inhibited insulin resistance, facilitated intestinal mucosal repair. Furthermore, TNVs rescued gut microbiota dysbiosis, restored the expression of key genes involved in glucose and lipid metabolism (ACC, AMPK, CD36, LXRα, PPAR-γ, SREBP-1), and activated the expression of glycolysis-related genes (G6Pase, GLUT2, PCK1, PEPCK) (12). Outer membrane vesicles (OMVs) released from garlic ELNs (GaELNs) trained human gut Akkermansia muciniphila (A. muciniphila) can reverse T2DM by increasing the GLP-1 plasma level (13). Similarly, targeted delivery carriers loaded with bioactive substances do not conform to the concept of traditional gut microbiome modulators. For instance, tartary buckwheat-derived ELNs (TB-ELNs) based dual-carriers are fabricated by loading chlorogenic acid (CGA) into the cores and bonding selenium nanoparticles (SeNPs) to the lipid membrane.TB-ELNs based dual-carriers are internalized by epithelial cells and transcytosis via the endoplasmic reticulum, thereby repairing the colonic physical barrier, regulating gut microbiota balance, and and synergistically controlling glucose metabolism (14).

It is traditionally believed that reciprocal host-microbiota interactions exist, but the complex interplay remains unclear. Recent multiple studies converge on a core principle: the host intestinal microenvironment influences the function and role of the microbiota. For example, *Akkermansia muciniphila (A. muciniphila)* is widely regarded as a “next-generation probiotic” that improves insulin sensitivity and lowers cholesterol. However, studies have shown that its effects are significantly dual-natured, as it can disrupt the mucosal barrier and exacerbate gut microbiota imbalance in acute radiation-induced intestinal injury (15, 16). Recent studies indicate that most solid tumors lacking natural cavity barrier damage and anatomical physical adhesion do not harbor independent microecology, and the stable colonization of microorganisms and their depth of effect on the host depend heavily on the physical space state (17). Whether it is Akkermansia, whose function reverses under pathological conditions, or tumor-associated microbiota whose distribution is determined by anatomical structure, it is evident that the host physical environment is the foundation for microbial colonization and function. Beyond the physical environment, gastrointestinal motility also serves as a basis for host-microbe interactions. Intestinal motility, as one of the oldest and most universal host traits, is a core evolutionary scaffold shaping microbial community structure. The flow velocity, shear force, and nutrient gradients generated by rhythmic host contractions directly select for microbial adhesion, biofilm formation, and metabolism (18). A clinical trial found that a high-fermentation diet can increase gut microbial diversity, but the newly classified microbiota did not originate from the fermented food itself. We speculate that this may be related to the improvement of host inflammatory levels by high-fermentation foods (19). Perhaps in actual treatment, it is necessary to fully consider the state of the host’s intestinal microenvironment. By altering the internal environment (e.g., physical environment), the structure and function of the gut microbiota can be directly or indirectly regulated, steering it toward a direction more beneficial to host health. This perspective also provides strong experimental support for the Envbiotics theoretical framework. Macroecological analysis confirms that one of the core characteristics of healthy individuals’ gut microbiota is the higher abundance and diversity of “uncultured bacteria” communities (20). Traditional microecological regulators focus on directly targeting known microbiota. However, for compounds that regulate such “uncultured bacteria” communities directly or indirectly by acting on the host, the classification framework of traditional microecological regulators is inadequate. The proposal of Envbiotics aims to cover substances that fall outside the scope of traditional classification frameworks.

In summary, Envbiotics not only represents an important supplement to the classification system of microecological preparations but also signifies a paradigm shift from “microbiota replacement therapy” to “host-microbiota interactions, ” providing a novel theoretical basis for developing new gut microbe-targeted intervention strategies for diabetes mellitus. However, the concept of Envbiotics still requires further refinement, and future research should focus on the standardized preparation of Envbiotics, its dose-response relationship, and its synergistic mechanisms with existing antidiabetic medications.
